# Precise single base substitution in the *shibire* gene by CRISPR/Cas9-mediated homology directed repair in *Bactrocera tryoni*

**DOI:** 10.1186/s12863-020-00934-3

**Published:** 2020-12-18

**Authors:** Amanda Choo, Elisabeth Fung, Isabel Y. Chen, Robert Saint, Peter Crisp, Simon W. Baxter

**Affiliations:** 1 School of Biological Sciences, University of Adelaide, Adelaide, SA Australia; 2 South Australian Research and Development Institute (SARDI), Adelaide, SA Australia; 3 Flinders University, Adelaide, SA Australia; 4 School of Agriculture, Food and Wine, University of Adelaide, Adelaide, SA Australia; 5 School of BioSciences, University of Melbourne, Melbourne, Australia

**Keywords:** Tephritids, Temperature sensitivity, Mutagenesis

## Abstract

**Background:**

Pest eradication using the Sterile Insect Technique (SIT) involves high-density releases of sterilized males that mate with wild females and ultimately suppress the population. Sterilized females are not required for SIT and their removal or separation from males prior to release remains challenging. In order to develop genetic sexing strains (GSS), conditional traits such as temperature sensitive lethality are required.

**Results:**

Here we introduce a known *Drosophila melanogaster* temperature sensitive embryonic lethal mutation into *Bactrocera tryoni*, a serious horticultural pest in Australia. A non-synonymous point mutation in the *D. melanogaster* gene *shibire* causes embryonic lethality at 29 °C and we successfully used CRISPR/Cas9 technology to recreate the orthologous *shibire* temperature sensitive-1 (*shi*^ts1^) mutation in *B. tryoni*. Genotypic analyses over three generations revealed that a high fitness cost was associated with the *shi*^ts1^ mutant allele and *shi*^ts1^ homozygotes were not viable at 21 °C, which is a more severe phenotype than that documented in *D. melanogaster*.

**Conclusions:**

We have demonstrated the first successful use of CRISPR/Cas9 to introduce precise single base substitutions in an endogenous gene via homology-directed repair in an agricultural pest insect and this technology can be used to trial other conditional mutations for the ultimate aim of generating genetic sexing strains for SIT.

**Supplementary Information:**

The online version contains supplementary material available at 10.1186/s12863-020-00934-3.

## Background

Queensland fruit fly, *Bactrocera tryoni* (Froggatt), can infest more than one hundred different host plants and is the most serious pest of horticulture in Australia [1]. Area wide integrated pest management programmes to control *B. tryoni* include the use of Sterile Insect Technique (SIT), which was first proposed by E. F. Kipling as a method of controlling insect populations [2]. SIT has been widely used to suppress or eradicate numerous pest species [3] through intensive releases of steriles into targeted locations. Sterilised males mate with wild females to produce non-viable embryos and reduce the population. Current practices for *B. tryoni* involve sterile releases of both males and females, yet the release of only sterilized males have proven to be more efficient for SIT of other tephritids [4]. The development of a *B. tryoni* genetic sexing strain (GSS) for conditional removal of females in SIT rearing facilities is highly desirable.

The generation of a functional GSS is a two-step process. First, an effective visual or inducible trait, such as embryonic temperature sensitive lethality, has to be identified or generated in the desired species. Second, male fitness must then be restored through translocation or insertion of a wild type allele onto the male Y-chromosome. Previous efforts to produce a *B. tryoni* GSS for SIT took advantage of a temperature sensitive lethal (tsl) mutation obtained through inbreeding laboratory cultures [5]. The *bent wings (bw)* strain carried a recessive curved wing mutation on chromosome 2, causing poor flight ability in addition to lethality at high temperatures. Heat treating 1 day old wild type or *bw* eggs at 40 °C for 4 h caused 4.6 and 80% lethality respectively, while rearing puparium at 31 °C caused 15% lethality in wild type and 100% lethality in *bw*. This *bw* tsl mutation was therefore proposed as a conditional lethal trait that could be used to form the basis of a *B. tryoni* GSS, although a tsl causing complete embryonic lethality at a temperature that does not result in a fitness cost for wild types would be more ideal. As the autosomal *bw* mutation affects both males and females, a functional *bw* allele (*bw*^+^) is required on the male Y chromosome to protect males from temperature sensitivity. Meats et al. [5] used gamma radiation on a strain without *bent wings* to induce translocations between chromosome 2 and the Y chromosome (2-Y translocations). Extensive screening through crossing viable irradiated lines with *bent wings* produced a strain where females expressed *bent wing* phenotype and males had normal wings due to the 2-Y translocation. Unfortunately, the 2-Y translocation males were also temperature sensitive and unsuitable for use as a GSS.

Random mutagenesis methods, including ionizing radiation, have been shown to induce genome wide germline mutations that often result in reduced fitness of the mutants [6–8]. Generating desirable mutants using these methods can therefore be challenging. Targeted approaches for precise gene editing and modification, including the Clustered Regularly Interspaced Short Palindromic Repeats (CRISPR)/CRISPR-associated 9 (Cas9) technology [9], provided new opportunities for creating GSS. The CRISPR/Cas9 technology can be used to induce frame-shift mutations in the form of indels (insertions or deletions) through the non-homologous end-joining (NHEJ) repair mechanism to knock out genes or introduce specific genetic modifications (“knock-in”) via the homology-directed repair (HDR) mechanism using a donor template. We have previously created a *B. tryoni* strain with a white eye phenotype, through a *white* gene knock out using the NHEJ pathway [10], demonstrating that the CRISPR/Cas9 technology is applicable to *B. tryoni* and could hence be used to produce a *B. tryoni* GSS. The generation of a functional GSS using CRISPR/Cas9 gene editing will require creating an efficient embryonic conditional lethal mutation such as a tsl mutation (usually through the HDR mechanism), followed by translocation or insertion of a functional copy of the gene into identified Y chromosome regions [11]. The aim of this study is to introduce a tsl mutation into the germline of *B. tryoni* using the CRISPR/Cas9 technology.

A GSS of the Mediterranean fruit fly (Medfly, *Ceratitis capitata* (Wiedemann)) that carries a tsl mutation causing complete embryonic lethality was previously generated using random mutagenesis [12]. Recreating the Medfly homologous mutation in *B. tryoni* using CRISPR/Cas9-mediated mutagenesis is currently not possible as its genetic basis is still unknown. An alternative approach is to take known temperature mutations characterised in other systems and introduce homologous mutations into the genome of *B. tryoni*. Studies have previously been conducted to identify tsl mutations in *Drosophila melanogaster* [13–16] and among them are those detected in the *shibire* gene. Shibire is a dynamin GTPase involved in formation of endocytic vesicles required for synaptic vesicle recycling and transmission at nerve terminals [17]. The *shibire* temperature sensitive-1 (*shi*^ts1^) mutation is a G-to-A point mutation that results in a single amino acid substitution at the boundary of the shibire GTPase domain [18]. The *shi*^ts1^ mutation results in embryonic lethality and adult paralysis when treated at the higher temperature of 29 °C [13]. The homologous mutation has also been shown to have a temperature sensitive effect in human cells [19], providing further support that this specific mutation results in a temperature sensitive phenotype.

Here we use the CRISPR/Cas9-mediated HDR pathway to successfully introduce a specific mutation with a donor template and the crRNA-tracRNA guide system, creating the orthologous *D. melanogaster shi*^ts1^ mutation in the *B. tryoni shibire* gene. We found the desired *shi*^ts1^ mutation to be homozygous lethal in *B. tryoni* even at ambient temperatures with a fitness cost to the heterozygotes, making it unsuitable as a genetic sexing trait. Nevertheless, our established CRISPR/Cas9 technique of generating precise single base substitution allows the possibility of introducing other candidate tsl mutations to obtain a functional *B. tryoni* GSS in the future.

## Results

### Identification of the *D. melanogaster shibire* ortholog in *B. tryoni*

**Fig. 1:**
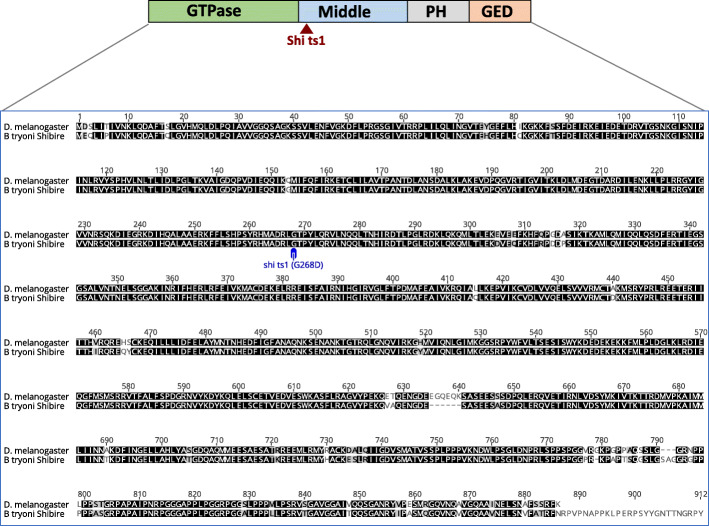
Protein alignment comparing the *D. melanogaster* and *B. tryoni* shibire. There is a high level of conservation between the two proteins (90% identity). Amino acid 268 is mutated in the *shi*^ts1^ mutant, which changes the glycine (G) residue to an aspartic acid (D) residue. Middle = Middle domain, PH = pleckstrin homology domain, GED = GTPase effector domain

A BLAST comparison search against the *B. tryoni* draft genome (JHQJ00000000) [20] was performed using the *D. melanogaster shibire* gene sequence (Flybase FBgn0003392), which identified an orthologous gene on scaffold Btry154 (GenBank accession JHQJ01000182.1 position 173,234….213,616). This scaffold has been mapped to one end of the *B. tryoni* chromosome 5 [21], which is homologous to the *D. melanogaster* X where the *D. melanogaster shibire* gene is located. A NCBI Conserved Domain search (CD-search) identified conserved domains in the *B. tryoni* shibire protein, including the domain where the *D. melanogaster shi*^ts1^ mutation is located (Fig. 1). The amino acid residue (Glycine-268) that causes temperature sensitivity in *D. melanogaster* when mutated to aspartic acid (the *shi*^ts1^ mutation) is conserved in the *B. tryoni* shibire ortholog (Fig. 1).

### Designing CRISPR/Cas9 components to target the *shi*^ts1^ locus in *B. tryoni*

**Fig. 2:**
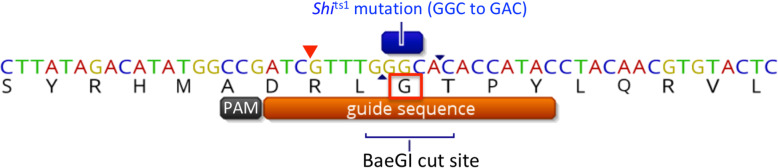
CRISPR guide sequence used to introduce the *shi*^ts1^ point mutation into *B. tryoni*. The single base change that is being induced in the *shi*^ts1^ locus is a ‘G’ to ‘A’, resulting in change of the amino acid residue glycine (G, highlighted by the red box) to aspartic acid and removing the BaeGI restriction site (GKGCM^C). The red arrow indicates the PAM cut site

A 635 bp region in exon 3 of the *shibire* gene was sequenced in eight *B. tryoni* individuals of the Ourimbah laboratory strain to determine if there were any single nucleotide polymorphisms (SNPs) within that region. A guide RNA sequence was designed with a protospacer adjacent motif (PAM) cut site seven bases upstream of the *shi*^ts1^ locus (Fig. 2). A blastn search of the guide RNA sequence against the *B. tryoni* reference genome revealed no likely off-target matches. The *shi*^ts1^ guide sequence had no additional exact hits and five other sequences contained at least 5 mismatches, but all lacked a PAM site.

**Fig. 3:**
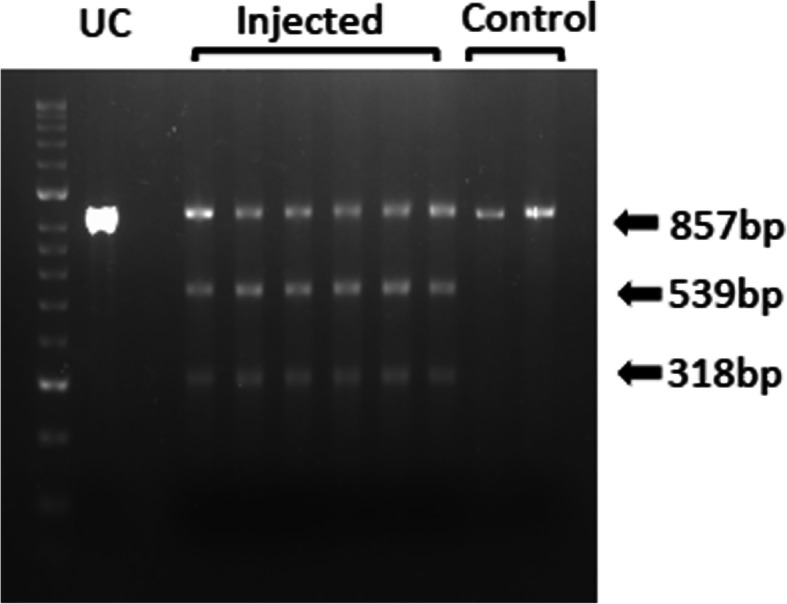
Assessing the efficiency of CRISPR/Cas9-mediated DNA cleavage at the *shi*^ts1^ locus. T7 Endonuclease I (T7EI) assay was performed to assess mutagenesis efficiency using the CRISPR/Cas9 RNP complexes. T7EI-induced cleavage at the *shi*^ts1^ target site (resulting in 539 bp and 318 bp fragments) was observed in all injected samples and not in the uninjected control embryos. This indicated successful targeting of the *shi* locus using that particular guide sequence. UC = uncleaved, injected sample was not treated with the T7E1

CRISPR/Cas9 mutagenesis efficiency using the *shibire* guide RNA sequence was assessed through a T7 Endonuclease I (T7EI) assay in vivo (Fig. 3, Additional File 2). The CRISPR/Cas9 ribonucleoprotein (RNP) complex was microinjected into 150–200 embryos less than one hour old, then after 24 h they were pooled into groups of 20–30 for DNA isolation and PCR amplification. Amplicon cleavage was observed for all injected samples in the T7EI assay, forming banding patterns consistent with indels introduced at the target site through CRISPR/Cas9 NHEJ. Amplicons from uninjected control embryos were not cleaved.

### Introduction of the *shi*^ts1^ mutation into *B. tryoni shibire*

**Fig. 4:**
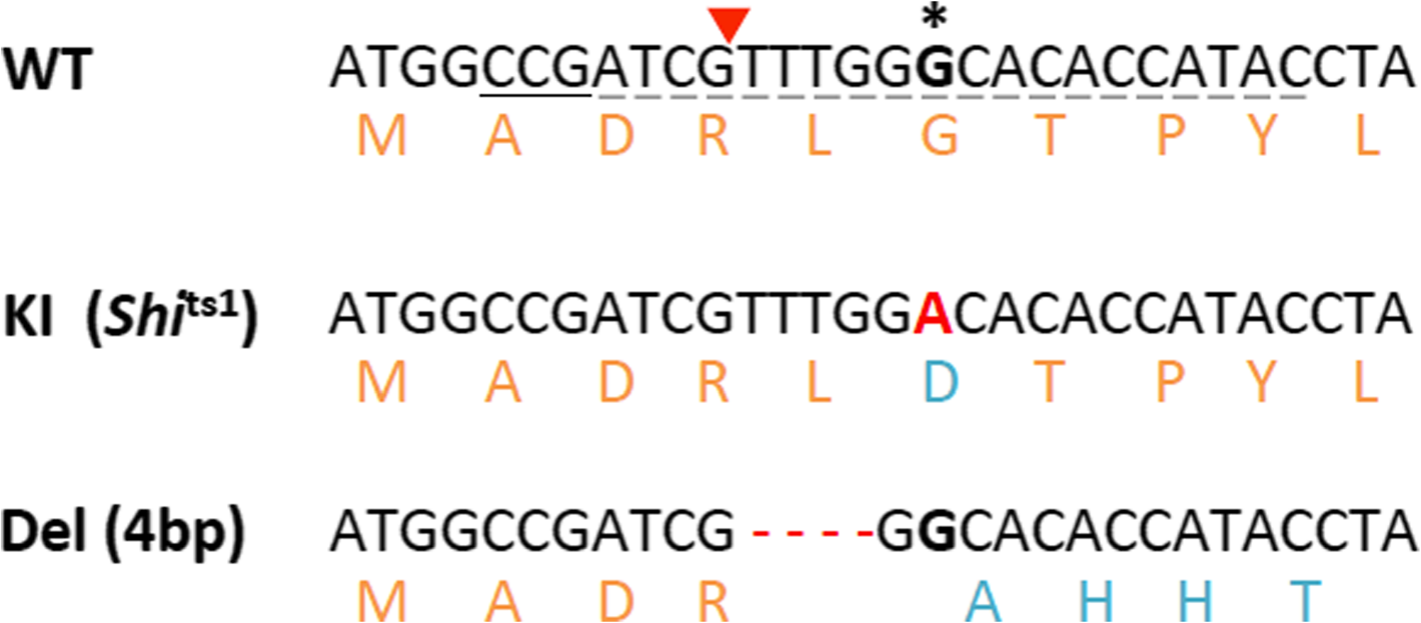
Mutant alleles identified from the G_1_ population at the *shi*^ts1^ locus. *Shi*^ts1^ CRISPR/Cas9 mutagenesis experiments produced two mutant genotypes (the *shi*^ts1^ single base substitution and a 4 bp deletion) in G_1_ progeny. The *shi*^ts1^ G to A mutant allele (highlighted in red) causes a glycine (G) to aspartic acid (D) amino acid change (highlighted in blue). A 4 bp deletion (indicated by the red dotted lines) causes a frameshift and consequently a change in the downstream amino acid sequence (highlighted in blue). WT = wild type (reference genome) sequence, KI = knock-in, Del = deletion. The 3 bp PAM sequence is underlined with a bold line and the 20 bp guide sequence with dotted lines, with the red arrow indicating the PAM cut site. The base where the *shi*^ts1^ point mutation is made is highlighted in bold and with an asterisk

In order to introduce the *shi*^ts1^ mutation into the *B. tryoni* genome, a 151 nt single-stranded oligo donor template (ssODN) containing the *shi*^ts1^ G➔A base substitution was designed (see Supplementary Table S1, Additional File 1). The CRISPR/Cas9 RNP complex consisting of the crRNA-tracrRNA complex and Cas9 enzyme was injected into embryos together with the ssODN HDR template. Three different concentrations of the ssODN (200 ng/μL, 250 ng/μL and 300 ng/μL) were tested to determine the optimal concentration for inducing mutagenesis. The ssODN concentration was found to inversely correlate with the percentage of injected embryo survival to adulthood (Table 1), suggesting that there is increased toxicity with higher ssODN concentrations. The 200 ng/μL ssODN concentration was found to be the optimal of these three concentrations, as it produced two germline mutants out of 14 G_0_ adults, including a female that carried the desired *shi*^ts1^ single base substitution (7.1% mutagenesis efficiency, Table 1, Fig. 4). Detection of the germline mutation was confirmed by genotyping G_1_ progeny from the individual mating of the G_0_ female with wild type laboratory males.

**Table 1:** Summary of the microinjections performed using the crRNA-tracrRNA RNP complex system to introduce the *shi*^ts1^ point mutation into *B. tryoni*

Injections	Concentration of ssODN	# embryos injected	# G_**0**_ adults (% survival)	# successful G_**0**_ matings	# G_**0**_ germline mutants^a^	Detected mutation	
						Mutation	# G_**1**_ mutants^b^
*Shi*^ts1^	200 ng/μL	254	14 (5.5%)	7	2 (14.3%)	Del (4 bp)	7 out of 12
						KI (*Shi*^ts1)^	18 out of 120
	250 ng/μL	473	10 (2.1%)	8	0	0	0
	300 ng/μL	310	5 (1.6%)	2	0	0	0

*Del* Deletion, *KI* Knock-in

^a^The mutagenesis efficiency is presented in brackets as the percentage of G_0_ flies with an identified germline mutation out of the total number of G_0_ adults obtained

^b^The number of G_1_ mutants identified out of a total of G_1_ progeny screened for that particular G_0_ germline mutant

### The *shi*^ts1^mutation carries a fitness cost in *B. tryoni*

**Fig. 5:**
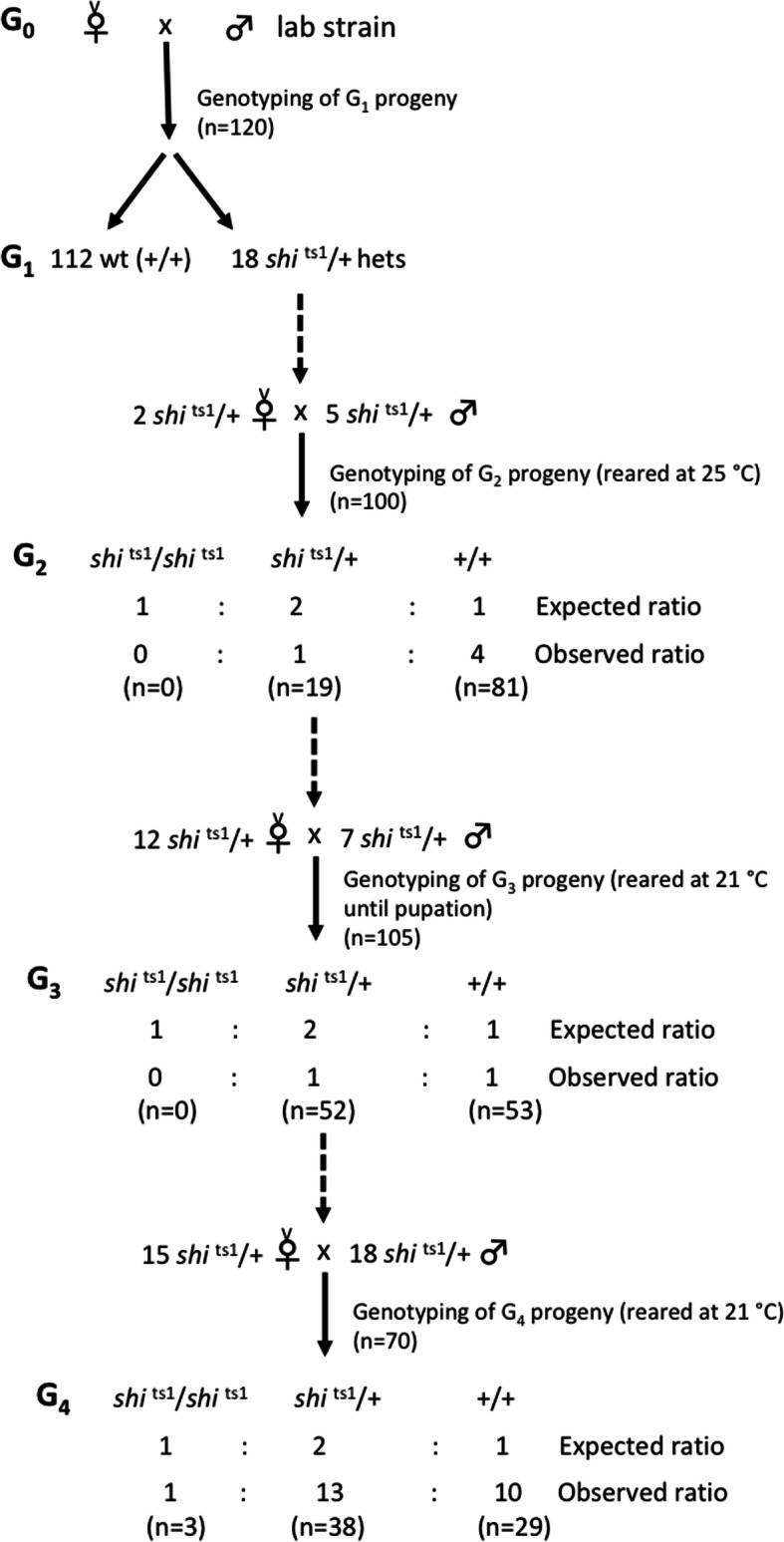
Genetic crosses performed to determine ratio of progeny carrying *shi*^ts1^ mutant allele(s). Crosses were carried out over three generations (G_1_-G_3_ matings) and the G_2_-G_4_ adult progeny were genotyped to determine if they carried any *shi*^ts1^ mutant allele(s). The observed ratio of progeny from each mating was compared to the expected ratio

Eighteen *shi*^ts1^/+ heterozygous mutants (11 males and 7 females) were identified from 120 G_1_ flies screened (Table 1). Seven of the surviving *shi*^ts1^/+ G_1_ flies (five males and two females) were mated to each other and the G_2_ progeny were reared at 25 °C (±2 °C). 100 G_2_ adult progeny were then genotyped with the expectation of Mendelian segregation ratios of 1:2:1 (*shi*^ts1^*/shi*^ts1^: *shi*^ts1^/+: +/+). However, no *shi*^ts1^*/shi*^ts1^ homozygotes were identified suggesting that this genotype is homozygous lethal at 25 °C (Fig. 5). Only 19 of the G_2_ flies were detected to be *shi*^ts1^/+ heterozygous mutants (7 males and 12 females) with the remaining 81 G_2_ flies wild type. This result deviates significantly (*X*^2^(2) =169.66, *p* < 2.2 × 10^− 16^) from the expected 1:2:1 ratio and highlights a fitness cost to *B. tryoni* individuals with at least one *shi*^ts1^ allele*.*

Homozygous *shi*^ts1^/*shi*^ts1^ *D. melanogaster* have been shown to have a 77% egg hatch rate at 22 °C and high pupal eclosion rate of 98% [22]. In order to determine whether *B. tryoni shi*^ts1^/ *shi*^ts1^ homozygotes are viable at a lower temperature similar to the *D. melanogaster* mutants, the 19 *shi*^ts1^/+ heterozygous G_2_ mutants were mated inter se and their eggs (G_3_ progeny) were collected and reared at 21 °C (±2 °C) until pupation, after which they were moved to 25 °C to increase development. Genotyping performed on 105 G_3_ adults revealed that there were again no *shi*^ts1^ homozygotes amongst the progeny (Fig. 5). 52 *shi*^ts1^/+ heterozygotes and 53 wildtype (+/+) flies were identified at 1:1 progeny ratio instead of the expected 2:1 ratio (*X*^2^(2) = 53.514, *p* < 2.4 × 10^− 12^). From the 52 *shi*^ts1^/+ heterozygotes identified in the G_3_ screening, 33 of the flies (18 males and 15 females) were then mated inter se*.* Eggs (G_4_ progeny) were collected from the mating and reared at 21 °C (±2 °C) this time until adult eclosion to determine the effects of rearing the eggs through to adulthood at the low temperature. Of the 70 G_4_ progeny genotyped, 29 were wild type flies, 38 were *shi*^ts1^/+ heterozygotes and 3 flies were identified as homozygous *shi*^ts1^/*shi*^ts1^flies. Although a small number of *shi*^ts1^/*shi*^ts1^ homozygotes was obtained when reared at 21 °C, they all died soon after genotyping. The deviation between observed (1:13:10) and expected (1:2:1) ratios was significant (*X*^2^(2) = 19.829, *p* < 4.9 × 10^− 5^). The continual failure to obtain viable *shi*^ts1^/*shi*^ts1^ homozygotes and *shi*^ts1^/+ heterozygotes at the expected proportions (25 and 50% of the total progeny respectively from each mating cross) demonstrated that there is lethality associated with the *shi*^ts1^ mutation in *B. tryoni* even at lower temperatures.

## Discussion

Targeted mutagenesis using the CRISPR/Cas9 genome editing technology has now been successfully achieved in a number of agricultural pest species, where non-specific indel mutations were generated through the NHEJ pathway [10, 23–28]. Whilst NHEJ-induced indel mutations have been useful for enabling the study of gene function, a greater focus has now been placed on using the HDR repair pathway particularly to introduce precise mutations to generate specific mutants without producing transgenic strains that contain DNA from other species. Conditional lethal mutants, such as temperature sensitive mutants, are crucial for development of effective SIT genetic sexing strains. Temperature sensitive mutations identified in the model organism *D. melanogaster* have primarily been found to be single base substitutions [13–16, 29], and precise CRISPR/Cas9-mediated HDR mutagenesis of homologous sites in *B. tryoni* may help generate these useful traits for producing a *B. tryoni* GSS. Successful HDR have been demonstrated in various dipteran species [30–34], involving the introduction or editing of transgenes, however there have been no reports of precise single base substitutions in endogenous genes.

The CRISPR/Cas9 system is a ribonucleoprotein (RNP) complex consisting of the bacterial endonuclease Cas9 and a guide RNA complex containing a unique sequence complementary to its target sequence in the genome [9]. The RNA complex can either be made up of two RNA structures – the CRISPR RNA (crRNA) and the trans-activating CRISPR RNA (tracrRNA) or a long single synthetic guide RNA (sgRNA) structure. Whilst a RNP complex consisting of the crRNA and tracrRNA mimics the bacterial system from which this CRISPR/Cas technology originates from, many research labs opt for the use of the sgRNA that combines the crRNA and tracrRNA into the single RNA structure and which can be easily synthesised in the laboratory [35].

We have previously demonstrated success using the in vitro-transcribed sgRNA method to generate indel mutations in the *B. tryoni white* gene via the NHEJ pathway [10] but have had no success using the same method to introduce a precise single base substitution through the HDR pathway (see Supplementary Methods, Table S2, Additional File 1). A recent study has shown that in vitro-transcribed sgRNAs, due to a triphosphate group introduced at the 5′ terminus by the T7 polymerase, can trigger the innate immune response in cells resulting in cell death [36]. This could potentially lead to poorer survival rates post microinjections in addition to the lethality induced by the microinjection process itself and consequently a larger number of injected embryos are required to obtain a desired mutant. That, coupled with the HDR pathway being the less efficient cellular repair pathway [37], could have contributed to difficulties in obtaining our desired mutant using the in vitro-transcribed sgRNA method in our *B. tryoni* organism. Some studies have shown that the use of crRNA-tracrRNA complexes can result in higher HDR efficiencies compared to sgRNAs [38–40]. There are also the benefits of the crRNAs and tracRNAs being easily chemically synthesised and modified, making the RNP complex more stable and less likely to induce an immune response, thus being an overall more efficient protocol [41]. In this study, we successfully utilised the crRNA-tracrRNA RNP complex system to introduce into the *B. tryoni* genome the specific *shi*^ts1^ single base substitution with a detected mutagenesis efficiency of 7.1%. This is, to our knowledge, not only the first report of CRISPR/Cas9-mediated precise single base substitution in an endogenous gene of an agricultural pest insect, but also the first report of gene editing using the crRNA-tracrRNA guide system in tephritids, which we have shown to be more effective than the commonly used sgRNA system in regards to making this precise *shi*^ts1^ base change.

In *D. melanogaster*, the *shibire* temperature lethal mutation *shi*^ts1^ was found to result in embryonic lethality and cause larval and adult paralysis if treated at 29 °C during the specific life stages [13]. We successfully engineered the homologous *shi*^ts1^ mutation into *B. tryoni* but were unable to obtain and/or maintain viable homozygotes even at 21 °C. A decrease in viability has been reported for *shi*^ts1^ homozygotes at 22 °C in *D. melanogaster,* with an overall 41% survival rate from the embryonic stage to eclosion [22], suggesting that the mutation does have a slight fitness cost; however in *B. tryoni*, the effects of the *shi*^ts1^ mutation on viability is far more severe. We found the *shi*^ts1^ mutation to be homozygous lethal even at low temperatures and resulted in significantly lower viability in the heterozygotes.

The *shi*^ts1^ mutation is thought to induce a conformational change in the protein, affecting the GTP binding site and protein aggregation, thus blocking endocytosis [42]. Despite Drosophilidae and Tephritidae diverging an estimated 70–100 million years ago [11, 43, 44], the shibire protein remains highly conserved. It is unclear why *B. tryoni shi*^ts1^ mutants appear to have a higher fitness cost than in *D. melanogaster*, however one possibility could be the contribution of modifier genes affecting shibire and/or its functional pathways, with different genetic modifiers present or with differential expression in *D. melanogaster* and *B. tryoni*. Indeed it has been observed that identical mutations in the same gene can lead to varying phenotypes in organisms with different genetic backgrounds [45]. This study highlights that replication of a phenotype may not always be successful despite high conservation of the protein sequence.

## Conclusions

We successfully introduced the *D. melanogaster* temperature sensitive lethal *shibire* ts1 (*shi*^ts1^) point mutation into the *B. tryoni* genome. The *shi*^ts1^ mutation was shown be homozygous lethal in *B. tryoni* and has a fitness cost in heterozygotes even at lower temperatures, making it unsuitable as a GSS. We have nonetheless demonstrated an efficient method for engineering precise single base mutations via the CRISPR/Cas9-mediated HDR pathway in *B. tryoni,* which can be applied to other tephritid flies and non-model organisms and be used in further efforts to generate a functional GSS for SIT.

## Methods

### Fly rearing

*Bactrocera tryoni* flies were from New South Wales Department of Primary Industries (NSW DPI), Ourimbah, Australia. Flies were reared under a controlled environment (25 ± 2 °C, 65 ± 10% relative humidity (RH) and under a 14:10 light/dark cycle), as described in Choo et al. [10]. Eggs from *B. tryoni* carrying the *shi*^ts1^ allele were maintained at 21 °C (±2 °C) unless described otherwise.

### *Bactrocera tryoni* shibire gene analyses

The *D. melanogaster shibire* gene sequence (FBgn0003392) and protein sequence (FBpp0290811) were obtained from Flybase and the gene sequence used in a blastn search against the *B. tryoni* genome (JHQJ00000000) with Geneious 7.17 and 10.1.3. Conserved domains within the *B. tryoni* shibire were identified through a NCBI Conserved Domain search (CD-search). Amplification of *B. tryoni* genomic DNA spanning the *shi*^ts1^ locus was performed as described in Choo et al. [10] using MyTaq polymerase, forward primer 5′-CGAGGATGAAACGGATCGTG-3′ and reverse primer 5′-GATCGTCAGGTCTGAAGTGC-3′ with the following cycling conditions: 95 °C for 2 min, 35 cycles of 95 °C for 15 s, 55 °C for 15 s and 72 °C for 3 min, followed by a final extension at 72 °C for 7 mins. Amplicon sequencing was carried out by the Australian Genome Research Facility (AGRF Adelaide).

### CRISPR/Cas9 reagents

All CRISPR/Cas9 reagents used to generate the *shi*^ts1^ mutant were obtained from Integrated DNA Technologies (IDT) as follows: purified Cas9 protein (Alt-R® S.p. Cas9 nuclease 3NLS, #1078729, 10 μg/μL), guide RNAs (customised Alt-R® CRISPR/Cas9 crRNA, 2 nmol and Alt-R® CRISPR/Cas9 tracrRNA, #1072532, 5 nmol) and customised ultramer® DNA oligo (4 nmol) which was used as the 151 nt single-stranded donor (ssODN) template for HDR. The guide RNAs were resuspended to a 100 μM stock solution with nuclease-free duplex buffer and the ultramer® DNA oligo to 1 μg/μL with ddH_2_O before use. The customised 20 bp crRNA sequence (*shi*^ts1^ – GTATGGTGTGCCCAAACGAT) was compared against the *B. tryoni* genome (JHQJ00000000) through a blastn search using Geneious 7.17 and 10.1.3 to verify that there are no likely off-targets.

### Embryo microinjections

Embryo microinjections were performed as described in Choo et al. [10] except for the steps stated otherwise. The injection mixes comprised of 0.3 μg/μL Cas9 protein, 0.12 μg/μL crRNA + 0.22 μg/μL rRNA, 0.2 μg/μL ssODN and 1x injection buffer (0.1 mM sodium phosphate buffer pH 6.8, 5 mM KCl) [46]. The crRNA and tracrRNA were first heated together at 95 °C for 5 min and cooled to room temperature to allow annealing to form a crRNA-tracrRNA complex. The crRNA-tracrRNA complex was then mixed with the Cas9 enzyme, ssODN and injection buffer to make the injection mix. Embryos 0–1 h old were adhered to a glass coverslip with Maruni rubber cement (No. 37022C) and injected under paraffin oil. Microscope slides with injected embryos were placed on 1% agar in a Petri dish, which was then placed in a “humid box” (a vented container with wet paper towels) and left for 48–72 h to allow larval hatching. Hatched first-instar larvae and developing embryos were then transferred to the gel diet for larval development and reared to adulthood, with all surviving adults designated as G_0_ flies.

### T7 endonuclease I cleavage assay

CRISPR/Cas9 mixes were microinjected into embryos less than 1 h old. Embryos that had not been injected were used as controls. After 24 h, embryos were pooled in groups of 20–30 and genomic DNA was isolated using the Phire Animal Tissue Direct PCR kit (#F140WH, ThermoFisher Scientific). PCR was performed to amplify 857 bp of the DNA region containing the shibire mutation loci using the Phire polymerase with the forward primer 5′-CACCAGTTTCGATGAGATCC-3′ and reverse primer 5′ CAAAGCTGAACCGGAACCTT-3′. The PCR cycling conditions used were 98 °C for 5 min, 35 cycles of 98 °C for 5 s, 60 °C for 5 s and 72 °C for 40 s, followed by a final extension at 72 °C for 1 min. 200 ng of PCR amplicon were then denatured and reannealed using the following conditions: 95 °C for 5 min, ramp down to 85 °C at − 2 °C/s and ramp down to 25 °C at − 0.1 °C/s. The annealed PCR products were then incubated with 1 μL of T7 endonuclease I (New England BioLabs, M0302S) at 37 °C for 15 mins. Products were visualised using 2% agarose (Scientifix Pty. Ltd., #9010E) gel electrophoresis.

### Molecular detection of CRISPR/Cas9-induced shibire tsl mutations

For the genotyping assays, genomic DNA was extracted from a single adult leg using the Phire Animal Tissue Direct PCR kit. PCR was performed as described above for the T7 Endonuclease I cleavage Assay. The PCR amplicons were then digested with the BaeG1 restriction enzyme (New England BioLabs, R0708S) at 37 °C for 1.5 h to distinguish potential heterozygous mutants (partially digested products) from wild type (digested products). PCR amplicons of heterozygotes were Sanger sequenced for genotype validation (AGRF Adelaide).

### Genetic crosses

Individual G_0_ flies were mated to 6–8 virgin flies from the Ourimbah laboratory strain. A minimum of 50 G_1_ adult progeny were collected from each successful G_0_ mating for genotyping, with the exception of when less than 50 G_1_ progeny were obtained. Mutagenesis efficiencies were calculated as the percentage of G_0_ adults with an identified germline mutation out of the total number of G_0_ flies obtained. Upon detection of a *shi*^ts1^ germline mutation, further crosses were carried out between identified *shi*^ts1/+^ heterozygous siblings of the G_1,_ G_2_ and G_3_ generations. Eggs were collected from each mating and reared under the conditions as described: G_2_ eggs reared to adulthood at normal rearing temperature (25 °C) while G_3_ and G_4_ eggs were reared at 21 °C up to pupation (and then moved to 25 °C) and adulthood respectively. Genotyping was performed on all obtained adult progeny. The ratios of G_2_ – G_4_ progeny carrying the *shi*^ts1^ mutant allele(s) were determined for the G_1_ – G_3_ matings and Pearson’s Chi-square goodness of fit tests were performed using R ‘chisq.test’ [47] to determine significance between the observed and expected genotypes.

## Supplementary Information

**Additional file 1.** Supplementary Methods. Supplementary Reference. Supplementary Table S1. Supplementary Table S2.

**Additional file 2.** Raw gel image of T7 Endonuclease I (T7EI) assay result.

## Data Availability

Data generated in this study is provided in the manuscript and supplementary information. Sequence data for the *shibire* gene was obtained from Genbank accession JHQJ01000182.1, position 173,234….213,616 and is available from https://www.ncbi.nlm.nih.gov/nuccore/JHQJ01000182.1 [20].

## Abbreviations

SIT: Sterile Insect Technique
GSS: Genetic Sexing Strain
tsl: Temperature sensitive lethal
CRISPR/Cas9: Clustered Regularly Interspaced Short Palindromic Repeats (CRISPR)/CRISPR-associated 9 (Cas9) technology
NHEJ: Non-homologous end-joining
HDR: Homology-directed repair
SNP: Single nucleotide polymorphism
PAM: Protospacer adjacent motif
T7E1: T7 Endonuclease 1
RNP: Ribonucleoprotein
ssODN: Single-stranded oligo donor
sgRNA: Synthetic guide RNA
crRNA: CRISPR RNA
tracrRNA: Trans-activating CRISPR RNA

## References

[1] Plant Health Australia (2016). The Australian handbook for the identification of fruit flies, Version 2.0.

[2] Knipling EF (1959). Sterile-male method of population control. Science.

[3] Klassen W, Curtis CF, Dyck VA, Hendrichs J, Robinson AS (2005). History of the sterile insect technique. Sterile insect technique principles and practice in area-wide integrated pest management.

[4] Rendón P, McInnis D, Lance D, Stewart J (2004). Medfly (Diptera: Tephritidae) genetic sexing: large-scale field comparison of males-only and bisexual sterile fly releases in Guatemala. J Econ Entomol.

[5] Meats A, Maheswaran P, Frommer M, Sved J (2002). Towards a male-only release system for SIT with the Queensland fruit fly, Bactrocera tryoni, using a genetic sexing strain with a temperature-sensitive lethal mutation. Genetica.

[6] Adewoye AB, Lindsay SJ, Dubrova YE, Hurles ME (2015). The genome-wide effects of ionizing radiation of mutation induction in the mammalian germline. Nat Commun.

[7] Blumenstiel JP, Noll AC, Griffiths JA, Perera AG, Walton KN, Gilliland WD (2009). Identification of EMS-induced mutations in Drosophila melanogaster by whole-genome sequencing. Genetics.

[8] Muller HJ (1928). The production of mutations by X-rays. Proc Natl Acad Sci U S A.

[9] Jinek M, Chylinski K, Fonfara I, Hauer M, Doudna JA, Charpentier EA (2012). Programmable dual-RNA-guided DNA endonuclease in adaptive bacterial immunity. Science.

[10] Choo A, Crisp P, Saint R, O'Keefe LV, Baxter SW (2018). CRISPR/Cas9-mediated mutagenesis of the white gene in the tephritid pest Bactrocera tryoni. J Appl Entomol.

[11] Choo A, Nguyen TNM, Ward CM, Chen IY, Sved J, Shearman D (2019). Identification of Y-chromosome scaffolds of the Queensland fruit fly reveals a duplicated gyf gene paralogue common to many Bactrocera pest species. Insect Mol Biol.

[12] Cáceres C (2002). Mass rearing of temperature sensitive genetic sexing strains in the Mediterranean fruit fly (Ceratitis capitata). Genetica.

[13] Grigliatti TA, Hall L, Rosenbluth R, Suzuki DT (1973). Temperature-sensitive mutations in Drosophila melanogaster. Mol Gen Genet.

[14] Mortin MA, Kaufman TC (1984). Development effects of a temperature-sensitive RNA polymerase II mutation in Drosophila melanogaster. Dev Biol.

[15] Pendleton RG, Rasheed A, Sardina T, Tully T, Hillman R (2002). Effects of tyrosine hydroxylase mutants on locomotor activity in Drosophila: a study in functional genomics. Behav Genet.

[16] Shellenbarger DL, Mohler JD (1978). Temperature-sensitive periods and autonomy of pleiotropic effects of l(1)Nts1, a conditional notch lethal in Drosophila. Dev Biol.

[17] Chen MS, Obar RA, Schroeder CC, Austin TW, Poodry CA, Wadsworth SC (1991). Multiple forms of dynamin are encoded by shibire, a Drosophila gene involved in endocytosis. Nature.

[18] van der Bliek AM, Meyerowitz EM (1991). Dynamin-like protein encoded by the Drosophila shibire gene associated with vesicular traffic. Nature.

[19] Damke H, Baba T, van der Bliek AM, Schmid SL (1995). Clathrin-independent pinocytosis is induced in cells overexpressing a temperature-sensitive mutant of dynamin. J Cell Biol.

[20] Gilchrist AS, Shearman DC, Frommer M, Raphael KA, Deshpande NP, Wilkins MR (2014). The draft genome of the pest tephritid fruit fly Bactrocera tryoni: resources for the genomic analysis of hybridizing species. BMC Genomics.

[21] Sved JA, Chen Y, Shearman D, Frommer M, Gilchrist AS, Sherwin WB (2016). Extraordinary conservation of entire chromosomes in insects over long evolutionary periods. Evolution.

[22] Poodry CA, Hall L, Suzuki DT (1973). Developmental properties of shibire ts1: a pleiotropic mutation affecting larval and adult locomotion and development. Dev Biol.

[23] Li J, Handler AM (2019). CRISPR/Cas9-mediated gene editing in an exogenous transgene and an endogenous sex determination gene in the Caribbean fruit fly, Anastrepha suspensa. Gene.

[24] Li F, Scott MJ (2016). CRISRP/Cas9-mediated mutagenesis of the white and sex lethal loci in the invasive pest. Drosophila suzukii Biochem Biophys Res Commun.

[25] Meccariello A, Monti SM, Romanelli A, Colonna R, Primo P, Inghilterra MG (2017). Highly efficient DNA-free gene disruption in the agricultural pest Ceratitis capitata by CRISPR-Cas9 ribonucleoprotein complexes. Sci Rep.

[26] Paulo DF, Williamson ME, Arp AP, Li F, Sagel A, Skoda SR (2019). Specific gene disruption in the major livestock pests *Cochliomyia hominivorax* and Lucina cuprina using CRISPR/Cas9. G3(Bethesda).

[27] Sim SB, Kauwe AN, Ruano REY, Rendon P, Geib SM (2019). The ABCs of CRISPR in Tephritidae: developing methods for inducing heritable mutations in the genera Anastrepha, Bactrocera and Ceratitis. Insect Mol Biol.

[28] Zheng W, Li Q, Sun H, Ali MW, Zhang H (2019). Clustered regularly interspaced short palindromic repeats (CRISPR)/CRISPR-associated 9-mediated mutagenesis of the multiple edematous wings gene induces muscle weakness and flightlessness in Bactrocera dorsalis (Diptera; Tephritidae). Insect Mol Biol.

[29] Suzuki D, Piternick LK, Hayashi S, Tarasoff M, Baillie D, Erasmus U (1967). Temperature-sensitive mutations in Drosophila melanogaster, I. relative frequencies among gamma-ray and chemically induced sex-linked recessive lethals and semilethals. Proc Natl Acad Sci U S A.

[30] Ahmed HMM, Hildebrand L, Wimmer EA (2019). Improvement and use of CRISPR/Cas9 to engineer a sperm-making strain for the invasive fruit pest Drosophila suzukii. BMC Biotechnol.

[31] Aumann RA, Schetelig MF, Hacker I (2018). Highly efficient genome editing by homology-directed repair using Cas9 protein in Ceratitis capitata. Insect Biochem Mol Biol.

[32] Basu S, Aryan A, Overcash JM, Samuel GH, Anderson MAE, Dahlem T (2015). Silencing of end-joining repair for efficient site-specific gene insertion after TALEN/CRISPR mutagenesis in Aedes aegypti. Proc Natl Acad Sci U S A.

[33] Gantz VM, Jasinskiene N, Tatarenkova O, Fazekas A, Macias VM, Bier E (2015). Highly efficient Cas9-mediated gene drive for population modification of the malaria vector mosquito Anopheles stephensi. Proc Natl Acad Sci U S A.

[34] Li J, Handler AM (2017). Temperature-dependent sex-reversal by a transformer-2 gene-edited mutation in the spotted wing drosophila, Drosophila suzukii. Sci Rep.

[35] Cho SW, Kim S, Kim JM, Kim J (2013). Targeted genome engineering in human cells with the Cas9 RNA-guided endonuclease. Nat Biotechnol.

[36] Kim S, Koo T, Jee HG, Cho HY, Lee G, Lim DG (2018). CRISPR RNAs trigger innate immune response in human cells. Genome Res.

[37] Rozov S, Permyakova NV, Deineko EV (2019). The problem of the low rates of CRISPR/Cas9-mediated knock-ins in plants: approaches and solutions. Int J Mol Sci.

[38] Aida T, Chiyo K, Usami T, Ishikubo H, Imahashi R, Wada Y (2015). Cloning-free CRISPR/Cas system facilitates functional cassette knock-in in mice. Genome Biol.

[39] Paix A, Folkmann A, Rasoloson D, Seydoux G (2015). High efficiency, homology-directed genome editing in Caenorhabditis elegans using CRISPR-cas9 ribonucleoprotein complexes. Genetics.

[40] Kotwica-Rolinska J, Chodakova L, Chvalova D, Kristofova L, Fenclova I, Provaznik J (2019). CRISPR/Cas9 genome editing introduction and optimization in the non-model insect Pyrrhocoris apterus. Front Physiol.

[41] Jacobi AM, Rettig GR, Turk R, Collingwood MA, Zeiner SA, Quadros RM (2017). Simplified CRISPR tools for efficient genome editing and streamlined protocols for their delivery into mammalian cells and mouse zygotes. Methods.

[42] Chen M, Green D, Liu L, Lam YC, Mukai L, Rao S (2002). Unique biochemical and behavioural alterations in drosophila shibire^ts1^ mutants imply a conformational state affecting dynamin subcellular distribution and synaptic vesicle cycling. J Neurobiol.

[43] Beverley SM, Wilson AC (1984). Molecular evolution in Drosophila and the higher diptera. II. A time scale for fly evolution. J Mol Evol.

[44] Kwiatowski J, Skarecky D, Bailey K, Ayala FJ (1994). (1994) phylogeny of Drosophila and related genera inferred from the nucleotide sequence of the cu, Zn sod gene. J Mol Evol.

[45] Kammenga JE (2017). The background puzzle: how identical mutations in the same gene lead to different disease symptoms. FEBS J.

[46] Choo A (2018). User method: Microinjection of *Bactrocera tryoni* (Queensland fruit fly) embryos. How to prepare Alt-R® CRISPR-Cas9 ribonucleoprotein complexes for microinjection.

[47] R Core Team (2018). R: A language and environment for statistical computing. R Foundation for Statistical Computing, Vienna, Austria.

