# Supplementary material for: Precise single base substitution in the shibire gene by CRISPR/Cas9-mediated homology directed repair in Bactrocera tryoni
Source: BMC Genet. 2020 Dec 18;21(Suppl 2):127. doi: 10.1186/s12863-020-00934-3 (PMC7747451; doi:10.1186/s12863-020-00934-3)
Supplement: Supplementary file 1 — Additional file 1. Supplementary Methods. Supplementary Reference. Supplementary Table S1. Supplementary Table S2. [file 12863_2020_934_MOESM1_ESM.docx]

**Additional File 1**

**Supplementary Methods**

Guide RNA (sgRNA) targeting the *shi*^ts1^ locus was generated via *in vitro* transcription using the Bassett and Liu (2014) method, as described in Choo *et al.,* 2018, using the following *shibire* exon3-specific oligonucleotide as the CRISPR F oligonucleotide (Qfly_shiCRISPR_ts1_F):GAAATTAATACGACTCACTATAGGT ATGGTGTGCCCAAACGATGTTTTAGAGCTAGAAATAGC. The 151nt single-stranded oligo donor (ssODN) was obtained from IDT as an Ultramer^®^ DNA oligo (4nmol) (Sequence in Table S1). Purified Cas9 protein was obtained from ToolGen (#TGEN_CP1) and ThermoFisher Scientific (B25641, 3μg/μL). Preparation of the ToolGen Cas9 protein and microinjection mixes were as described in Choo *et al*. (2018) with various Cas9, sgRNA and ssODN concentrations used (Table S2).

**Supplementary Reference**

Bassett A, Liu JL. CRISPR/Cas9 mediated genome engineering in Drosophila. Methods 2014;69(2):128-36.

**Supplementary Tables**

Table S1. Sequences of the single-stranded donor oligos (ssODN) used as the donor template.

| **Mutation** | **ssODN sequences (5’-3’)** |
| --- | --- |
| *Shi*^ts1^ | TCACGCAAACCTGGAAGCGTATCACGAATATGATTGGTCAACTGTTGATTGAGTACACGTTGTAGGTATGGTGTGTCCAAACGATCGGCCATATGTCTATAAGATGGATGACTAAGGAAGAATTTGCGTTCAGCCGCCAAGGCTTGATGAA |

Table S2. Summary of the microinjections performed using the *shi*^ts1^ sgRNA method

| **CRISPR/Cas9 injection mix concentrations (ng/μL)** | | | **# embryos injected** | **# G_0_ adults (% survival)** | **# successful G_0_ matings** | **# G_0_ germline mutants*** | **Detected mutation** | |
| --- | --- | --- | --- | --- | --- | --- | --- | --- |
| **Cas9** | **sgRNA** | **ssODN** |  |  |  |  | **Mutation** | **# G_1_ mutants^** |
| 300 | 280 | 200 | 217 | 4 (1.8%) | 4 | 1 (25%) | Del (16bp) | 2 out of 24 |
| 300 | 280 | 300 | 109 | 3 (2.8%) | 3 | 0 | 0 | 0 |
| 300 | 850 | 300 | 393 | 8 (2.0%) | 7 | 0 | 0 | 0 |
| 600 | 280 | 300 | 95 | 1 (1.0%) | 0 | 0 | 0 | 0 |
| 900 | 280 | 200 | 180 | 4 (2.2%) | 1 | 0 | 0 | 0 |
| 900 | 280 | 300 | 174 | 1 (0.5%) | 1 | 0 | 0 | 0 |

* The mutagenesis efficiency is presented in brackets as the percentage of G_0_ flies with an identified germline mutation

out of the total number of G_0_ adults obtained.

^ The number of G_1_ mutants identified out of a total of G_1_ progeny screened for that particular G_0_ germline mutant.

Del = deletion
