# Supplementary figures and images for: Precise single base substitution in the shibire gene by CRISPR/Cas9-mediated homology directed repair in Bactrocera tryoni
Source: BMC Genet. 2020 Dec 18;21(Suppl 2):127. doi: 10.1186/s12863-020-00934-3 (PMC7747451; doi:10.1186/s12863-020-00934-3)

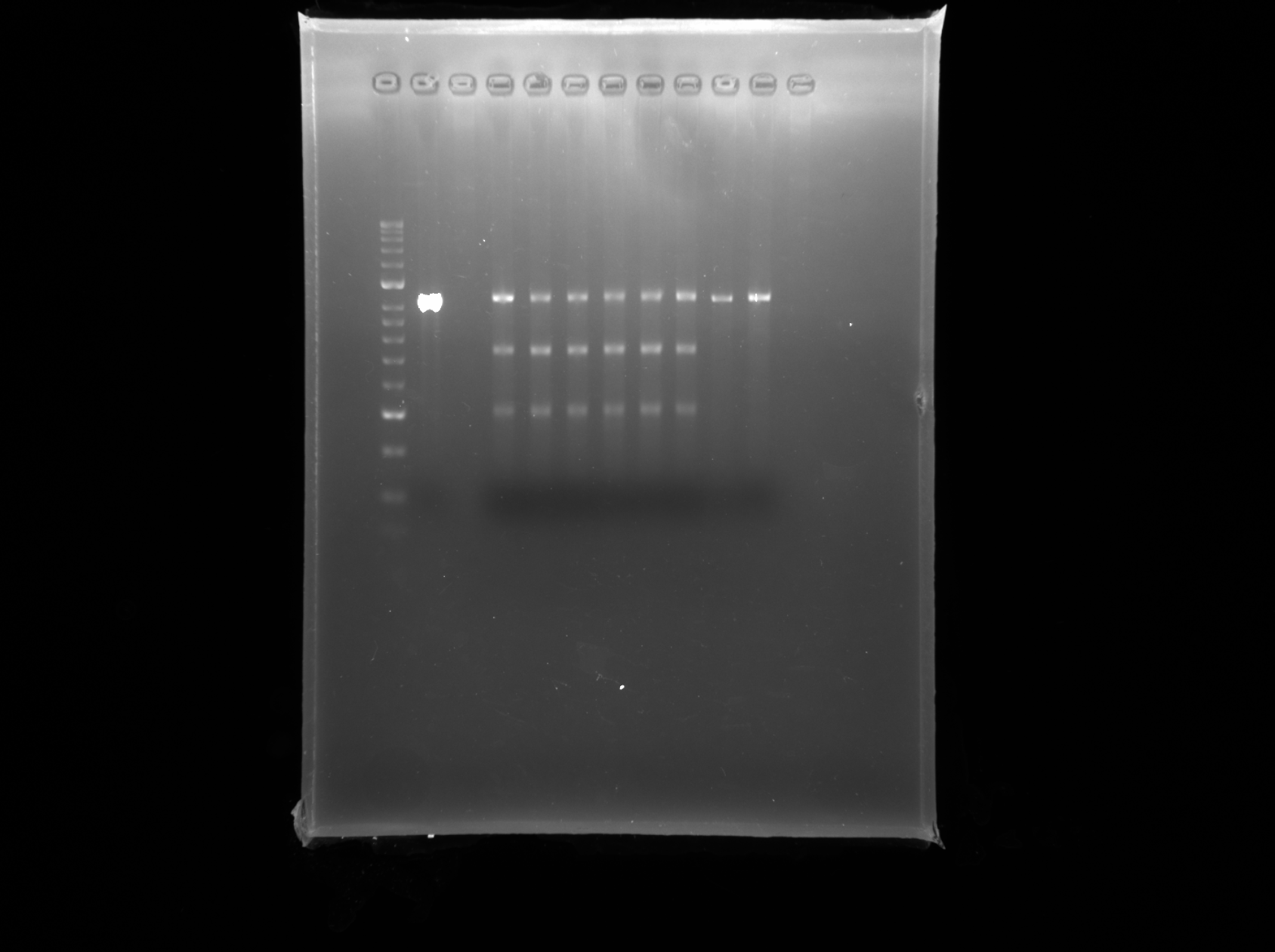

Supplement: Supplementary file 2 — Additional file 2. Raw gel image of T7 Endonuclease I (T7EI) assay result. [file 12863_2020_934_MOESM2_ESM.tif]
